# Altools: a user friendly NGS data analyser

**DOI:** 10.1186/s13062-016-0110-0

**Published:** 2016-02-17

**Authors:** Salvatore Camiolo, Gaurav Sablok, Andrea Porceddu

**Affiliations:** Università degli studi di Sassari, Dipartimento di Agraria, SACEG, Via Enrico De Nicola 1, Sassari, 07100 Italy; Plant Functional Biology and Climate Change Cluster (C3), University of Technology Sydney, PO Box 123 Broadway, NSW 2007 Sydney, Australia

**Keywords:** Next-generation sequencing, Copy number variation, SNPs, Indels, Large deletions, Re-sequencing

## Abstract

**Background:**

Genotyping by re-sequencing has become a standard approach to estimate single nucleotide polymorphism (SNP) diversity, haplotype structure and the biodiversity and has been defined as an efficient approach to address geographical population genomics of several model species. To access core SNPs and insertion/deletion polymorphisms (indels), and to infer the phyletic patterns of speciation, most such approaches map short reads to the reference genome. Variant calling is important to establish patterns of genome-wide association studies (GWAS) for quantitative trait loci (QTLs), and to determine the population and haplotype structure based on SNPs, thus allowing content-dependent trait and evolutionary analysis. Several tools have been developed to investigate such polymorphisms as well as more complex genomic rearrangements such as copy number variations, presence/absence variations and large deletions. The programs available for this purpose have different strengths (e.g. accuracy, sensitivity and specificity) and weaknesses (e.g. low computation speed, complex installation procedure and absence of a user-friendly interface). Here we introduce Altools, a software package that is easy to install and use, which allows the precise detection of polymorphisms and structural variations.

**Results:**

Altools uses the BWA/SAMtools/VarScan pipeline to call SNPs and indels, and the dnaCopy algorithm to achieve genome segmentation according to local coverage differences in order to identify copy number variations. It also uses insert size information from the alignment of paired-end reads and detects potential large deletions. A double mapping approach (BWA/BLASTn) identifies precise breakpoints while ensuring rapid elaboration. Finally, Altools implements several processes that yield deeper insight into the genes affected by the detected polymorphisms. Altools was used to analyse both simulated and real next-generation sequencing (NGS) data and performed satisfactorily in terms of positive predictive values, sensitivity, the identification of large deletion breakpoints and copy number detection.

**Conclusions:**

Altools is fast, reliable and easy to use for the mining of NGS data. The software package also attempts to link identified polymorphisms and structural variants to their biological functions thus providing more valuable information than similar tools.

**Reviewers:**

This article was reviewed by Prof. Lee and Prof. Raghava.

**Open peer review:**

Reviewed by Prof. Lee and Prof. Raghava. For the full reviews, please go to the Reviewers’ comments section.

**Electronic supplementary material:**

The online version of this article (doi:10.1186/s13062-016-0110-0) contains supplementary material, which is available to authorized users.

## Implementation

### Background

Genome-based polymorphic scans are the standard method to establish the degree of conservation and phylogenetic imprinting among the related plant taxa. Approaches based on re-sequencing have recently been exploited for the discovery of single nucleotide polymorphisms (SNPs) and insertion/deletion polymorphisms (indels) as a proxy for the phyletic patterns of evolution [1]. In addition to the creation of SNP maps, it is useful to identify SNPs associated with particular traits in order to localize quantitative trait loci (QTLs) suitable for molecular breeding programs [2].

In the last decade, the optimization of next-generation sequencing (NGS) chemistry and platforms has increased the throughput of sequencing while reducing costs. Although the generation of large amounts of sequence data is no longer a bottleneck in scientific investigations, the interpretation of the data remains challenging. Re-sequencing approaches produce millions of short reads 50–400 bp in length, although the latest technologies are likely to yield longer reads. When a target genome (TG) is re-sequenced, the alignment of such reads to a reference genome (RG) results in the detection of sequence variants such as SNPs and indels, and several alignment algorithms have been developed to detect them [3]. NGS platforms also generate sequencing errors, so other tools have been developed to reduce the number of false polymorphisms by introducing suitable statistical tests [4].

Although many aligners such as BWA [5] and Bowtie [6] incorporate algorithms that identify SNPs and indels quickly and accurately, they fail to detect large genomic deletions (hundreds to thousands of bases) possibly due to the segmental duplication of the genome and the retro-transposition of short and long interspersed elements (SINES and LINES) [7]. These types of polymorphisms are better highlighted by software that detects anomalous insert sizes in the alignment of paired-end reads, or by long-read sequencing approaches [8]. Alternatively, splitting each read into two portions can identify reads spanning the deleted segment (e.g. the deletion breakpoints) [9]. Tools such as Pindel [10], Breakdancer [11] and PEMer [12] rely on such strategies to identify large deletions, and must deal with the compromise between speed and the accuracy of breakpoint detection. Inferring the deletion coordinates from the distance between two mapped paired-end reads is inaccurate because the insert size is usually part of a distribution rather than a precise value. The identification of split-mapped reads is also an extremely time consuming and computationally demanding task.

Resequencing data have also been used to detect large genomic rearrangements such as copy number variations (CNVs) and presence/absence variations (PAVs) [13]. CNVs reflect duplication or deletion events that change the copy number of specific genomic sequences when comparing target and reference genomes. Alignment coverage at each reference position will increase in a duplicated segment and decrease in a deleted segment, so the depth of coverage (DOC) is often used to identify CNVs [13]. PAVs are identified by detecting reference positions that are not covered by any target genome reads.

Computational tools for sequence alignment and analysis are often difficult to install and use, particularly for non-specialist researchers with limited experience in the field of bioinformatics. Here we present Altools, a user-friendly software platform for the interpretation of resequencing data. The pipeline helps the user to achieve the alignment of sequenced reads against a reference genome, the discovery of SNPs/indels (at the genomic and transcript levels), CNVs, PAVs and large deletions through an intuitive graphical user interface (GUI). The algorithms included in Altools (Additional file 1: Figure S1) ensure the rapid and accurate analysis of sequence data and produce informative statistics that link the sequence data to biological functions [14].

### Materials and methods

#### Sequence data

*Arabidopsis thaliana* reference genome (Col0 ecotype) together with the corresponding gene annotation file was downloaded from the TAIR website (ftp://ftp.arabidopsis.org/home/tair/Genes/TAIR7_genome_release/). Gff2sequence [15] was used to generate FASTA formatted sequences of coding sequences (CDS) and untranslated regions (UTR). Resequencing data for the Tsu1 and Bur0 genotypes were downloaded from the SRA database (http://www.ncbi.nlm.nih.gov/sra/) (Additional file 2: Table S1).

#### Genome simulation

The R package RSVSim [16] was used with default parameters to generate *A. thaliana* simulated genomes that included deletions and duplications (maxDups = 10) of variable sizes (2000, 10,000 and 50,000 bp). For such rearranged genomes, dwgsim software (http://davetang.org/wiki/tiki-index.php?page=DWGSIM) was used to simulate Illumina paired-end 70-bp reads at different coverages (parameters: −C *cov* -c 0 -S 2 -e 0.0001-0.01 -E 0.0001-0.01, with *cov* equal to 4, 10, 20, 40 and 100). The same tool was used to generate simulated 70-bp paired end reads for the original *A. thaliana* genome with 40x coverage.

#### Evaluation of polymorphism quality

We applied the positive predictive value (PPV) and sensitivity tests to determine the robustness of SNPs and indels. The PPV is the portion of the total number of called polymorphisms that are correct [17]. Sensitivity indicates the ratio between the number of correctly called polymorphisms and the total number of genuine polymorphisms [17]. PPV and sensitivity were also used to evaluate the reliability of predicted large deletions and duplications. In this case, the number of positions included in the identified structural variants was divided by either the total number of bases in each structural variant (PPV) or by the total number of bases representing genuine structural variants (sensitivity).

#### Read alignment: mapping raw reads against a reference genome

The Read alignment tool allows the user to map a set of FASTQ-formatted reads to a reference genome using BWA [5] as the aligner, to sort and index the alignment file with SAMtools [18] and to call statistically significant polymorphisms with VarScan [19]. BWA was preferred over other aligners because it performs better than similar tools (e.g. Bowtie2) when analysing longer reads [20] (a scenario that will become more common for future sequencing technologies). Similarly, VarScan was chosen because of its high sensitivity [21] and better performance in lower-coverage sequencing runs [22]. Both tools have been implemented in Altools without modifications and therefore their performance has not changed. Altools will automatically recognize paired-end and single-end datasets and align them accordingly. Edit distance, number of threads (thus allowing for parallel computing) and any additional BWA flags can be specified by the user. When the alignment of reads is complete, a pileup-formatted file is generated by SAMtools [18] considering only those alignments that fulfil specific user-defined requirements (“minimum alignment quality”, “minimum base quality” and “additional pileup parameters” in the GUI). More information can be found in the Altools manual provided with the software.

#### Pileup analyser: providing faster access to the alignment data

The Pileup analyser tool is used to generate a pileup folder containing files related to each chromosome in the reference genome. Only information about position, reference genome nucleotide, target genome nucleotide, coverage and presence/absence of SNPs and indels is reported in such files, with the aim of reducing disk space usage and data processing times during further analysis. Pileup analyser also offers several configurable filter settings relative to the minimum number of reads, the base quality, the minimum p-value and threshold allele frequency for calling SNPs and indels. A comprehensive summary statistics file is also produced, reporting the percentage of non-covered chromosomes, the frequency of SNPs and indels, specific coverage of bases G|C and A|T, and the frequency of bases involved in selected polymorphisms.

#### Coverage analyser: detecting CNVs and PAVs

The Coverage analyser tool is designed to investigate CNVs and PAVs based on the local depth of coverage. Anomalous coverage values may reflect the structure of the target genome (i.e. duplications may be present in the reference genome), so CNV detection requires that alignment data from both the target and reference genomes are compared. Coverage analyser initially calculates the average coverage for the reference genome (RG_avCov_) and target genome (TG_avCov_) while computing only informative positions (i.e. coverage >0). A series of adjacent windows is then generated along the chromosomes, and for the *i*^*th*^ window an average coverage is calculated for both the reference genome (RG_windCov(i)_) and the target genome (TG_windCov(i)_) by computing the information reported in the relative pileup folders. Genomic portions that feature TG_windCov(i)_ = 0 but RG_windCov(i)_ >0 are immediately reported in the output as “zero coverage” regions, which highlight potential PAVs. Furthermore, for each *i*^*th*^ window, the value ρ_(i)_ is calculated as the ratio between the average coverage of the target and reference genomes in that window:

$$ \rho (i) = \frac{T{G}_{WindCov(i)}}{R{G}_{WindCov(i)}} $$

The DNAcopy algorithm [23] is then used to split the DNA into segments featuring homogeneous values of ρ_(i)_ (hereafter ρ_seg_). For each segment *j*, this value is normalized in order to account for the average coverage of the two segments:

$$ {\rho}_{seg Norm(j)} = {\rho}_{seg(j)}\ \frac{R{G}_{avCov}}{T{G}_{avCov}} $$

Moreover, for each segment, the average coverage of the target genome (TG_segCov(j)_) and reference genome (RG_segCov(j)_) are also calculated. Coverage analyser then reports losses and gains according to the following rationale: for the *j*^*th*^ segment, the hypothetical copy number for both the reference and target genomes is calculated by dividing the segment average coverage by the overall average coverage:

$$ T{G}_{segCopy(j)} = \frac{T{G}_{segCov(j)}}{T{G}_{avCov}}\kern3.25em R{G}_{segCopy(j)} = \frac{R{G}_{segCov(j)}}{R{G}_{avCov}} $$

If one or more copies of segment *j* have been lost from the target genome then the following relationship should be satisfied:

$$ T{G}_{segCopy(j)}\ \le R{G}_{segCopy(j)}-1 $$

However, if one considers a diploid organism that loses a segment copy in only one of the homologous chromosomes, the following relationship is more accurate:

$$ T{G}_{segCopy(j)}\ \le R{G}_{segCopy(j)}-0.5 $$

The above can be reformulated as:

$$ {\rho}_{segNorm(j)}\ R{G}_{segCopy(j)}\ \le\ R{G}_{segCopy(j)}-0.5 $$

This leads to the conclusion that a segment can be defined as lost if the following relationship is satisfied:

$$ {\rho}_{segNorm(j)\_ loss}\ \le\ 1-\frac{0.5}{R{G}_{segCopy(j)}} $$

Similarly, a gained segment is reported if the following relationship is satisfied:

$$ {\rho}_{segNorm(j)\_ gain}\ \ge\ 1+\frac{0.5}{R{G}_{segCopy(j)}} $$

DNAcopy allows the merging of segments whose ρ_seg_ values are at least three standard deviations apart, therefore creating a smoothed dataset. Coverage analyser also performs the search for lost and gained segments on such datasets. Importantly, Coverage analyser not only returns the coverage ratio but also the individual calculated copy number for both the reference and target genomes. This feature provides a deeper insight into the meaning of the ratio value (e.g. a value of 2 may derive from a 2:1 or 4:2 ratio, among others).

#### Sliding analysis: visualizing coverage and polymorphism data

The Sliding analysis tool computes the average coverage together with the frequency of SNPs and indels within either adjacent or sliding windows along the chromosome. Both the raw data and the corresponding plots are generated, so this tool quickly highlights highly polymorphic regions or sites potentially containing CNVs.

#### Large deletions finder: fast identification of deletions breakpoints

Common aligners that use short reads are not suitable for the detection of long deletions. The Large deletions finder tool uses a folder containing SAM-formatted files that are produced following the alignment of paired-end reads to a reference genome. A deletion is called when the mapping distance between two mate-reads is higher than a user-defined threshold. Overlapping deletions can be merged if the distance between the first mate for both sets of paired ends does not exceed a user-defined number of nucleotides. Altools returns the approximate coordinates of the deletion boundaries at this stage (Additional file 3: Figure S2A). An additional alignment step is performed using BLASTn to precisely identify the deletion breakpoints. Two ranges are defined that are 2000 nucleotides wide and centred on the approximate start and end positions, respectively (Additional file 3: Figure S2B). All read pairs for which at least one mate is mapped within such ranges are extracted from the SAM-formatted alignment file and mapped onto the reference genome by BLASTn alignment. Reads that did not map onto the reference genome originally, possibly due to a broken alignment, will produce hits that can be used to infer the real deletion boundaries (Additional file 3: Figure S2C).

Coverage analyser carries out an additional test to highlight potential false positive deletions reflecting intrachromosomal duplication events. The first 200 nucleotides beyond the upstream deletion breakpoint are extracted from the reference genome and used again as a BLASTn query to search for additional alignments. In the output file, further fields are reported for each deletion indicating the position of these secondary alignments, their percentage of identity and alignment coverage. We define deletions that feature such supplementary fields such as ambiguous, as explained in more detail in the Altools manual (Additional file 4: Figure S3). Finally, the coverage of the deleted regions is reported in order to speculate whether the detected structural variation is homozygous or heterozygous, and to test for the presence of the deleted regions at other positions within the target genome.

#### Polymorphism analyser: linking variants to biological functions

When SNPs and indels have been identified using the BWA/SAMtools/VarScan pipeline, the Polymorphism analyser tool can be used to highlight those nucleotide variations that affect the genic portions, i.e. coding sequences (CDS) and untranslated regions (UTR). This tool requires the pileup folder, an additional folder containing FASTA-formatted CDS and UTR sequences, and the gff3-formatted gene annotation file. Polymorphism analyser returns a table that reports information such as: (a) the genic portion of the sequence (CDS, 3UTR and/or 5UTR), (b) the gene name (c) the relative position of the polymorphism, (d) the nucleotides called in the reference genome and in the aligned reads, (e) the zygosity of the mutation, (f) amino acid substitutions due to non-synonymous SNPs, including mutations generating a premature stop codon, and (g) any frameshift caused by indels within the CDS.

#### Alignment comparison

The 1:1 Alignment tool compares the pileup folders of two different alignments on the same reference genome and reports the common and unique polymorphisms.

#### Gene extractor

The Large deletion finder and Coverage analyser tools feature an option to generate a GE file that can be analysed in more detail using the Gene Extractor tool. The latter also requires a gff3-formatted annotation file and returns a list of genes that are partially (marked with the flag 0) or totally (marked with the flag 1) included within a selected structural variation.

### Performance

#### SNP/indel identification in simulated genomes

The *A. thaliana* genome (TAIR7) was used as a scaffold to generated five sets of paired-end Illumina reads with 4x, 10x, 20x, 40x and 100x coverage, respectively. For each coverage dataset, reads were aligned to the original reference genome using the Reads alignment tool with default parameters. The Pileup analyser tools was then used (see Additional file 5: Table S2 for settings) to detect the simulated polymorphisms. Although the PPVs were >0.99 for each of the analysed datasets, sensitivity increased to a plateau at 20x coverage for both SNPs and indels (Table 1). Moreover, whereas the SNP calling sensitivity reached a maximum value of 0.98, indel identification was poor with a maximum value of 0.81 at 40x coverage.

**Table 1:** Performance of the Altools platform (detection of polymorphisms). Statistical analysis of Altools polymorphism calling was carried out at five simulated coverage levels

Coverage	4x	10x	20x	40x	100x
dgwsim generated polymorphisms	121,388	122,074	121,368	121,540	121,638
dgwsim generated SNPs	107,054	107,411	106,766	107,372	107,277
dgwsim generated indels	14,334	14,663	14,602	14,168	14,361
Altools total called SNPs	35,714	81,647	102,493	105,164	105,580
Altools correctly called SNPs	35,650	81,482	102,274	104,910	105,243
Altools false positive SNPs	64	165	219	254	337
Altools total called indels	3049	8307	11,134	11,542	11,657
Altools correctly called indels	3040	8280	11,112	11,503	11,621
Altools false positive indels	9	27	22	39	36
PPV					
SNPs	1.00	1.00	1.00	1.00	1.00
Indels	0.33	0.76	0.96	0.98	0.98
Sensitivity					
SNPs	1.00	1.00	1.00	1.00	1.00
Indels	0.21	0.56	0.76	0.81	0.81

#### Structural variation identification in simulated genomes

**Fig. 1:**
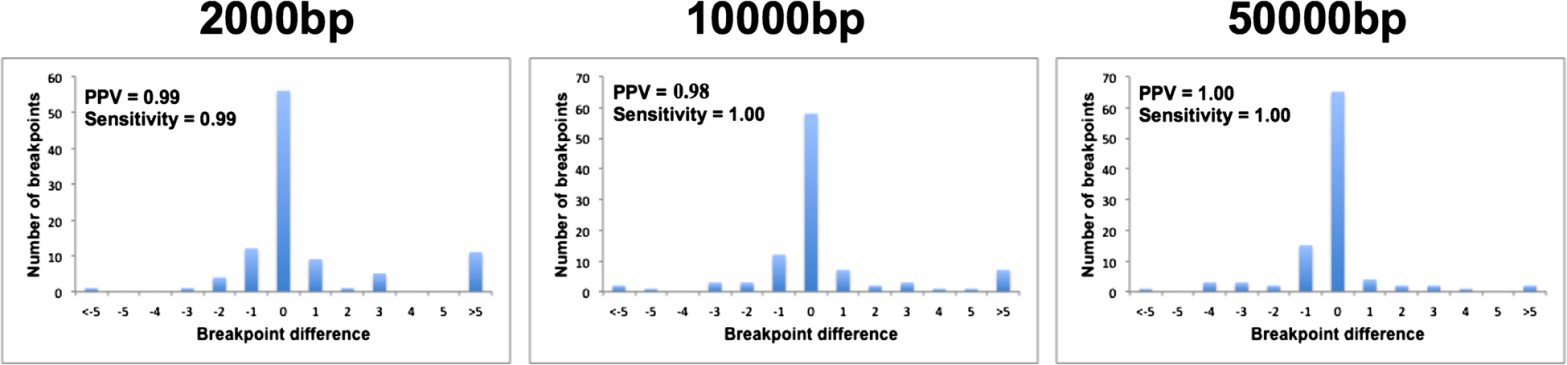
Performance of the Large deletion finder tool (detection of large deletion breakpoints). Distribution of the differences between detected and expected breakpoint positions called by the Large deletion finder tool together with the corresponding PPV and sensitivity. The plots represent the results on simulated read datasets with 10x coverage and three large deletion sizes (2000, 10,000 and 50,000 bp)

Fifty deletions of 2000 bp were introduced into the *A. thaliana* genome and the resulting simulated sequence was used to generate five sets of paired-end Illumina reads with 4x, 10x, 20x, 40x and 100x coverage, respectively. The same test was then repeated by simulating 10,000 and 50,000 bp deletions. The Large deletions finder tool was used to localize the simulated deletions in each dataset. The PPV and sensitivity were >0.97 for all the datasets and in many cases they reached their maximum value (Figs. 1 and Additional file 6: Figure S4). Furthermore, we computed the distribution of the differences between the observed and simulated breakpoints. The median was 0 at all parameters for coverage and deletion size, with differences of a few nucleotides between the 10^th^ and 90^th^ distribution quartiles (Fig. 1 and Additional file 6: Figure S4). The Large deletions finder tool was compared to the widely-used Pindel software [10] and the former showed superior performance in terms of execution time and, in most cases, also PPV and sensitivity (Additional file 7: Table S3).

We also simulated 50 duplications of 2000 bp in the same reference genome and generated five sets of paired-end Illumina reads with 4x, 10x, 20x, 40x and 100x coverage, respectively. The approach described above was used to investigate duplications of 10,000 and 50,000 bp. In each of the simulated datasets, the maximum number of duplications was 10. Coverage analyser was used to localize the duplicated regions and determine the number of copies based on a reference genome pileup folder derived from the alignment and pileup of *A. thaliana* simulated reads. A 50-bp window was used and only losses/gains larger than 500 bp were sent to the output file.

**Fig. 2:**
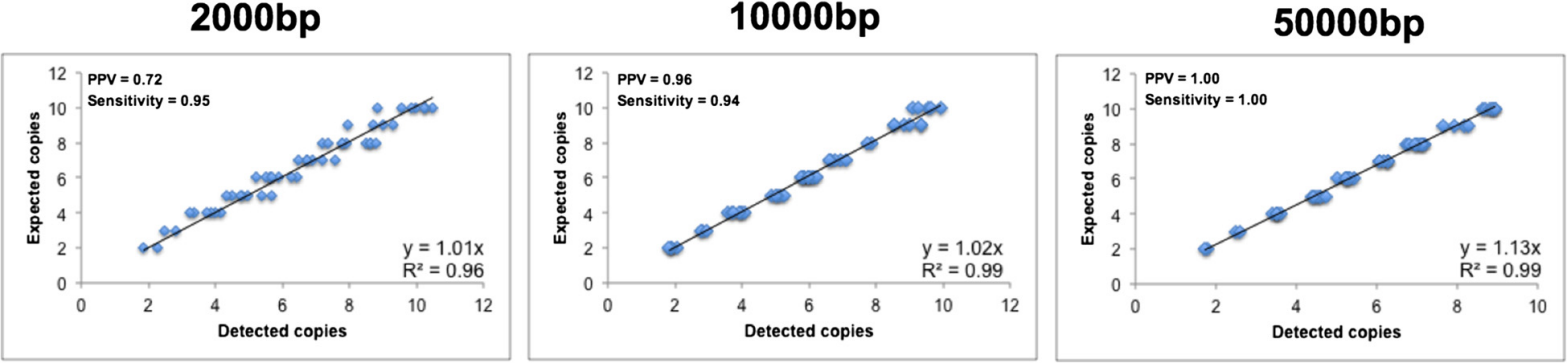
Performance of the Coverage analyser tool (detection of copy number variation). Scatterplot showing differences between detected and expected copy numbers called by the Coverage analyser tool together with the corresponding values of PPV and sensitivity. The plots represent the results on simulated read datasets with 10x coverage and three duplication sizes (2000, 10,000 and 50,000 bp)

The software achieved the best performance when only large duplications were present, resulting in the highest PPVs (0.97–1) and sensitivities (0.99–1) as shown in Figs. 2 and Additional file 8: Figure S5. However, the sensitivity declined to ~0.95 for the duplications of 2000 and 10000 bp, although the PPV was poor only for the 4x simulated dataset (PPV_2000bp_ = 0.21, PPV_10000bp_ = 0.65) as shown in Additional file 8: Figure S5. The copy number was also predicted precisely, with the slope between the detected and expected copy numbers always higher than 0.9 (Figs. 2 and Additional file 8: Figure S5). The comparison of this module with other software for the detection of CNVs, e.g. CNVseq [24], confirmed its excellent performance in terms of execution times, PPV and sensitivity (Additional file 7: Table S3).

#### Analysis of *A. thaliana* resequencing data using Altools

Altools was used to analyse the real resequencing data of two *A. thaliana* accessions (Bur0 and Tsu1) for the robust detection of polymorphisms and to estimate the scalability of the approach. The Pileup analyser tool identified several key features, such as: (a) a higher coverage of G|C compared to A|T bases (Additional file 9: Table S4), which is a known bias for some Illumina sequencing platforms [25]; (b) a higher frequency of polymorphisms in chromosome 4 (Additional file 10: Figure S6); and (c) maintenance of the genomic structure despite the SNP and indel events (Additional file 11: Figure S7).

The Polymorphism analyser tool highlighted the presence of 133,129 SNPs and 5343 indels within the CDS and UTRs of Bur0 transcripts. Interestingly, 94 % of the SNPs we identified were homozygous, compared to only 61.2 % of the indels (Table 2). The higher degree of SNP homozygosity reflects the status of *A. thaliana* as an autogamous plant species, whereas the different zygosity ratio in the context of indels suggests they are less likely to become fixed due to their potential deleterious effects, e.g. frameshifts in CDS or regulatory disruption in the UTRs. SNPs in the CDS resulted in 49,369 amino acid substitutions, 573 premature stop codons and the loss of the stop codon in at least one allele of 114 genes (Table 2). A similar picture emerged when the Tsu1 resequencing data were analysed, although the SNP frequency proved to be more homogenous when comparing the CDS and UTRs in this accession (~0.29 %).

**Table 2:** Polymorphisms found in the genomes and transcripts of *A. thaliana* accessions Bur0 and Tsu1

		Bur0	Tsu1
# Homozygous SNPs		125,234	107,257
# Heterozygous SNPs		7895	7203
# Homozygous indels		3271	2514
# Heterozygous indels		2072	1677
	CDS	0.32	0.28
SNP frequency	3utr	0.36	0.29
	5utr	0.36	0.29
	CDS	0.003	0.003
Indel frequency	3utr	0.059	0.045
	5utr	0.063	0.049
# Amino acid mutations		49,369	43,215
# Premature stop codons		573	469
# Lost stop codons		114	101

The 1:1 Alignment tool was used to compare Bur0 and Tsu1 polymorphisms, revealing that nearly 30 % of the polymorphisms were common to both accessions (Additional file 12: Figure S8). The Coverage analyser tool was used to investigate loss and gain events in Bur0 by comparing its resequencing data to the *A. thaliana* simulated data (accession Col0) as previously described (window size = 50, minimum number of windows to merge = 4, minimum structural variant size = 1000 bp). Nearly 4.4 million bp were shown to be lost from the Bur0 genome, whereas 3.4 million bp were gained (Table 3). Gene Extractor was used to investigate whether such structural variations could include annotated genes. Although the identified structural variants comprised more than 6 % of the *A. thaliana* genome, only a few hundred genes were totally included in the corresponding regions (Table 3). A gene ontology (GO) singular enrichment analysis (SEA) using the web-based server Agrigo (http://bioinfo.cau.edu.cn/agriGO/analysis.php) revealed that the gained genes were mostly involved in the respiration pathway (Additional file 13: Table S5) whereas the missing genes (lost and zero coverage) were enriched in stress-response functions (Additional file 14: Table S6).

**Table 3:** Coverage analyser results for *A. thaliana* accession Bur0. Total number of bases detected as gains, losses and zero coverage areas together with the number of annotated genes found in these areas

	Total length (bp)	# Included genes
Gains	3,429,100	145
Losses	4,443,400	116
Zero coverage	4,406,500	155

## Discussion

In this paper we present Altools, a new software pipeline for the analysis and interpretation of NGS data. Altools features a GUI-enabled workflow for variant calling that guides the user through all steps, beginning with reference-assisted alignment and ending with the functional annotation of identified variants. Altools relies on a Java-built GUI that provides a user-friendly bioinformatics environment together with several algorithms developed in C++ that maximize the computational performance. Although many software platforms have been developed to handle NGS data analysis, Altools offers a unique set of advantageous features. The BWA/SAMtools/VarScan pipeline is used for the alignment and identification of SNPs and indels, and to the best of our knowledge this is the first time these components have been embedded a single software platform and the overall performance has been verified. We found that the proposed strategy achieved satisfactory results in terms of PPV and sensitivity, although the best performance was achieved at coverages of 10x or more (Table 1). The performance and scalability of the workflow was equivalent to or in some cases even better than other available tools [17]. The sensitivity detection was better for SNPs than indels (Table 1). This may reflect the low edit distance used in the alignment step (BWA flag –n = 4) which can reduce the probability of alignment for reads featuring longer insertions or deletions.

A new algorithm was developed for the identification of large deletions. This takes into account paired-end reads mapping on the same chromosome but at a distance that is incompatible with the expected insert size, and this can determine the approximate coordinates of large deletions. The BLAST algorithm is then used to accurately detect the deletion breakpoints by using the broken alignment of reads spanning the identified deletions. Two additional features make the Large deletion finder tool superior to similar tools. First, coverage of the deleted segment is also calculated in the reference genome. This can provide a deeper insight on the typology of the lost DNA portion, i.e. the presence of aligned reads within deletions may reflect either a heterozygous structural variation or the presence of a paralogous region elsewhere in the genome. Second, the Large deletion finder tool also tests whether the deletion flanking regions are duplicated in additional positions of the chromosome. This feature, together with the number of reads supporting the structural variation, allowed us to exclude potential false positive deletions and achieve good performance in terms of PPV, sensitivity and precision of breakpoint detection for all the simulated datasets we analysed (Figs. 1 and Additional file 6: Figure S4).

The Coverage analyser tool achieved satisfactory PPV and sensitivity values together with a precise calculation of the copy number in most of the simulated datasets (Figs. 2 and Additional file 8: Figure S5). The performance was poorer when we analysed datasets featuring lower coverage and smaller duplicated segments because the method is sensitive to random coverage fluctuations that are more easily averaged in longer segments.

One of the main advantages of Altools is its ability to link SNPs, indels, CNVs, PAVs and large structural variations with biological outcomes. The benefit of this approach emerged from the analysis of two *A. thaliana* accessions, Bur0 and Tsu1. First, Pileup analyser produced statistics that were used for the assessment of the sequencing quality (e.g. G|C vs A|T coverage) while revealing that small polymorphisms (SNPs and indels) preserve the general AT-rich nucleotide composition profile (Additional file 11: Figure S7). Because this tool considers single chromosome datasets, chromosome 4 was identified as the most polymorphic in both accessions (Additional file 10: Figure S6).

The Coverage analyser tool allowed the identification of CNVs and PAVs in the Bur0 accession and revealed that almost 6 % of the reference genome is involved in such structural variations. Nevertheless, the Gene extractor tool showed that only a few hundred annotated genes were included completely within the detected CNVs and PAVs as expected, and that most structural variations were intergenic (or non-annotated) sequences. Interestingly, GO enrichment revealed ontologies associated with the respiration pathway (Additional file 13: Table S5) which corresponds to the ability of Bur0 shoots to produce larger amounts of several sugars compared to the Col0 accession under specific conditions [26]. The analysis of CNVs and PAVs also showed that many of the genes that have been lost from the Bur0 accession are related to stress-response functions (Additional file 14: Table S6) matching the more stress-sensitive characteristics of Bur0 compared to Col0 [27].

The Polymorphism analyser tool allowed the identification of genes in which SNPs or indels caused gene loss, premature truncation or amino acid substitutions. A simple evaluation of polymorphism frequencies within transcripts showed how SNPs are more likely than indels to become fixed in the CDS, with indels featuring much less frequently in the CDS compared to the UTRs. This hypothesis was confirmed by the higher percentage of heterozygous indels, contrasting with the autogamy of *A. thaliana* (Table 2). Finally, polymorphisms in the Bur0 and Tsu1 accessions were compared to find common and unique SNPs and indels, an additional Altools feature that could be used to investigate phylogenetic relationships, develop a DNA barcoding system or conduct genome wide association studies.

## Conclusions

Advances in the NGS technologies in the last years have led to the development of streamlined workflows for the analysis and interpretation of NGS data. In this context, Altools offers a unique combination of features including an intuitive GUI, a straightforward installation procedure and user-friendly menus suitable for researchers with only basic informatics skills. The new algorithm for the identification of several types of structural variations was fast, accurate and sensitive, equalling or exceeding the performance of contemporary software platforms. Finally, the Altools pipeline is not solely based on the comparative analysis of sequencing data but also the biological interpretation of complex datasets.

## Availability and requirements

Project name: Altools

Project home page: http://sourceforge.net/projects/altools/

Operating system: Linux 64bit

Programming language: Java, C++, R

Other requirements: xterm, R package DNAcopy, Java version 1.8.0_45 or later.

License: GNU GPL

Any restriction to use by non-academics: no restriction applied

## Reviewer’s comments

### Reviewer’s report 2: Prof. Sanghyuk Lee

Reviewer recommendations to authors:

Following points needs to be addressed for improving the quality of the work. 1. Most of pipelines lack an objective comparison with other tools publicly available. For example, they implemented BWA/samtools/Varscan for identifying SNPs and indels and it showed satisfactory performance in terms of PPV and sensitivity in their simulation study. However, its performance should be compared with other programs such as GATK utilities, PINDEL, Scalpel. CNVs are identified with their own in-house developed algorithm. Again, its performance should be compared with other tools for similar purposes (e.g. XHMM, ExomeDepth, Conifer, CONTRA, and exomeCopy). Without such comparison, it is difficult to judge whether Altools’ result are superior to those tools and nobody would use the tool. 2. The pipeline is tightly designed with very limited flexibility. Better approach would be to allow users to choose proper tools and processes like the GALAXY workflow engine. New and better tools are constantly released and users should be able to choose such updated tools if necessary. I believe that there exist better tools than Varscan in variant calling. Furthermore, the hard-wired pipeline of Altools is difficult to modify. For example, it is usually recommended to incorporate adaptor trimming, duplicate removal, and alignment recalibration for pre-processing of the NGS data in analyzing well-established model organisms. 3. The packing of tools needs significant improvement. I do not feel that the tool is really user-friendly with poor flexibility, no utility tools for log or process management, and no unique visualization support.

Minor issues:

English editing is strongly recommended.

Authors’ response to reviewer 2: *We would like to thank Professor Lee for his valuable suggestions. Please find hereafter a point by point response to the raised concerns.*

Major revisions.

We ran a benchmark test on Altools by comparing its performance with that of CNVseq for the detection of CNVs and Pindel for the detection of large deletions. The results (Additional file 7: Table S3) show that our software performed better in terms of execution time and, in general, in terms of PPV and sensitivity. The choice of the BWA aligner and VarScan polymorphism caller is now better explained in the text. We also appreciated the suggestion to improve the GUI by including a utility for log or process management, a visualization tool and a wider collection of aligners, polymorphism callers and read pre-processing tools and we intend to consider these suggestions for future Altools updates. For the time being, we believe that relying on widely-used file formats such as SAM, BAM and SAMtools pileup will already deliver a certain degree of flexibility to the Altools environment. For example, users can apply their favourite tools to generate compatible files and can still submit their data to the Altools structural variation detection algorithm.

Minor issues.

A professional scientific editing service has carried out a thorough revision of the manuscript.

### Reviewer 2’s comments to the revised manuscript:

As suggested in the previous review, authors compared the performance of Altools with CNVseq for CNVs and Pindel for large indels, and report better PPV and sensitivity. However, I think that the comparison target programs were not properly chosen. Both CNVseq and Pindel were published in 2009 and I believe that many other programs have been published for the same purpose. Furthermore, the issue of limited flexibility was not resolved yet. Even though Altools can be combined with various file formats in principle, experts with such capability would not use a pipeline tool not supporting recent advanced algorithms.

Authors’ response: *We would like to thank Professor Lee for his comments. Although we are aware of the most recent algorithms for the identification of polymorphisms and structural variations, we decided to benchmark Altools against Pindel and CNVseq because these software platforms are widely used, their quality is well established, and comparative tests against similar tools have been published in the recent literature (*e.g. *J. Zhang et al., 2014, Horticulture Research 1:14045; D. H. Ghoneim, 2014, BMC Research Notes 7:864, J. Duan, 2013, PlosOne 8:e59128). Indeed Professor Lee suggested Pindel as one of the platforms we should use for comparison. Finally, as indicated in our previous response, we are already working to improve the flexibility of Altools and compatibility with more recent algorithms will be introduced in a forthcoming update.*

### Reviewer’s report 3: Prof. Gajendra Raghava

Reviewer recommendations to authors:

In this manuscript, a pipeline developed for analyzing NGS data has been described. This is important pipeline for researchers working in the filed of genomics. In the present form this manuscript is not publishable as authors have not justified their claims. In addition selection of tools integrated in this manuscript need to be justified. Major comments 1. In past number of pipelines have been developed on NGS, author should show comparison of Altools with existing tools. 2. Authors claim that their pipeline is fast (fast in terms of what?)). In order to justify their claim they should benchmark their method in term of execution time used to process NGS data. 3. In addition, authors should show superiority of individual tools integrated in their pipeline over existing tools. This is important to show application of this pipline. 4) Altools pipeline contains eight major modules or components, author should list indigenous and third party software separately. Graphical flowchart of Altools would be useful for readers to understand components of the pipeline.

Minor issues:

1) This manuscript need to be revised thoroughly as it contain several grammatical and typographical mistakes. (e.g. genome wise association (GWAS) studies should be genome-wide association studies (GWAS). This pipeline has been mentioned Altools and ALtools in manuscript, it should be uniform 2) Additional file 11: Figure S7 is mentioned at page 14 (Line 41), which is otherwise missing. 3) In Table 2, what is the meaning of values having comma in between, e.g. 0,003? 4) In Table 1; they show total called and true called and false called SNPs. What about missed SNPs, which were generated by dgwsim software, but not called at all by Altools? 5) owtie was not used while it can take care of splice variants? Preference for BWA over Bowtie should be mentioned somewhere. 6) There is need to generate comprehensive manual for Altools

Author’s response to reviewer 3: *We would like to thank Prof. Raghava for his exhaustive review. Please find hereafter a point by point response to the raised concerns*

Major revisions.

1. Altools was benchmarked against two published software platforms for the determination of copy number variations (CNVs) and large deletions. The results (Additional file 7: Table S3) show that our software performed better in terms of execution time and, in general, in terms of PPV and sensitivity.
2. The execution speed is now reported and compared to similar software platforms (Additional file 7: Table S3).
3. The choice of the different software modules is now better explained in the text.
4. A flowchart illustrating the original and third-party software within Altools has been added to the revised version of the manuscript.

Minor issues

1. A professional scientific editing service has carried out a thorough revision of the manuscript. This included the careful standardization and correction of all software names, the checking of abbreviations and initialisms for accuracy, grammatical corrections and style revision.
2. The missing figure has now been added.
3. “,” has been replaced by “.” as decimal separator in all the tables.
4. The sensitivity values were calculated as “the fraction of simulated variants which were called from the sequence data” (ref 17) and is intended to address the concern raised by the reviewer.
5. The preference for BWA over Bowtie2 as the aligner is now addressed in the revised manuscript
6. A comprehensive manual for Altools is included in the software folder.

## Additional files

Additional file 1: Figure S1. Flowchart describing the eight Altools modules. Blue portions represent novel algorithms, whereas red portions represent third-party embedded software. (DOC 21 kb) Additional file 2: Table S1. Sequence read archive (SRA) experiments for *A. thaliana* accessions Bur0 and Tsu1 available at http://www.ncbi.nlm.nih.gov/sra. (DOC 209 kb) Additional file 3: Figure S2. Pipeline for the identification of deletion breakpoints. (a) Approximate deletion boundaries are inferred by detecting mapped paired-end reads that align at a distance that is not compatible with the expected insert. Overlapping sets of improperly-mapped mates (e.g. possibly underlining the same deletion) are merged at this stage. (b) A 2000-bp range is selected in the reference genome at each of the found deletion boundaries (deletion start ± 1000 bp and deletion end ± 1000 bp). Reads that are mapped within these regions are extracted from the alignment file together with the corresponding unmapped mates. (c) BLASTn is used to map reads identified at point (b) onto the reference genome and deletion breakpoints are inferred by the position of the detected partial alignments. (DOC 21 kb) Additional file 4: Figure S3. Possible duplication interference affecting the correct identification of a large deletion. In a real deletion, reads mapping to the genomic portion A have their mates mapped to portion B at a distance that is not compatible with their library insert size. However, if a deletion did not occur between A and B, but rather B is duplicated somewhere upstream within the same chromosome, then reads mapping to A may have their mates mapped either in B or in Bdup. Mate pairs aligning in the portions A–Bdup will feature a mapping distance that is not compatible with their insert and, in this case, a deletion may be erroneously called. (DOC 21 kb) Additional file 5: Table S2. Pileup analyser parameters to detect the simulated polymorphisms in the *A. thaliana* genome with different reference coverage values. (DOC 207 kb) Additional file 6: Figure S4. Distribution of the differences (PPV and sensitivity) between detected and expected breakpoint positions derived from Large deletion finder analysis of the simulated reads dataset (coverage 4x, 20x, 40x and 100x) with three large deletion sizes (2000, 10000 and 50000 bp). (DOC 21 kb) Additional file 7: Figure S5. Scatterplot showing differences (PPV and sensitivity) between detected and expected copy numbers calculated by the Coverage analyser tool on simulated reads datasets (coverage 4x, 20x, 40x and 100x) and three duplications sizes (2000, 10000 and 50000 bp). (DOC 21 kb) Additional file 8: Table S3. Benchmark of Altools for the detection of copy number variations (CNVs) and large deletions. The Coverage analyser module was compared to CNVseq [23] by testing its performance on the simulated *A. thaliana* genome with 10x coverage and three CNV segment sizes (2000, 10,000 and 50,000 bp). Default parameters were used in CNVseq except the window size (−−window-size 50) for the sake of uniformity with the Altools settings. The Large deletions finder module was compared to Pindel [10] by testing its performance on the simulated *A. thaliana* genome with 10x coverage and three deleted segment sizes (2000, 10,000 and 50,000 bp). To compare the software platforms under equivalent conditions, Pindel was set to output only deletions (−r false -t false -l false) while setting all the remaining parameters to their default values (for the detection of 50,000-bp deletions the flag –x 6 was added). Benchmarking was carried out on a server equipped with an Intel(R) Xeon(R) CPU X5660 working at 2.80 GHz. (DOC 21 kb) Additional file 9: Table S4. G|C bias in the Bur0 and Tsu1 Illumina NGS datasets. (DOC 21 kb) Additional file 10: Figure S6. Frequency of (A) SNPs and (B) indels in the alignment of Bur0 and Tsu1 sequences on the *A. thaliana* reference genome. (DOC 21 kb) Additional file 11: Figure S7. (Top) Frequency of the four nucleotides in the reference and target genomes at a polymorphic site. (Bottom) Frequency of the four nucleotides among the inserted and deleted bases. (TIFF 142 kb) Additional file 12: Figure S8. Comparison of polymorphisms (SNPs and indels) found in the *A. thaliana* accessions Bur0 and Tsu1. (TIFF 68 kb) Additional file 13: Table S5. Gene Ontology enrichment analysis of the Bur0 accession transcripts that are enclosed in gained regions (P = process and F = function). (DOC 21 kb) Additional file 14: Table S6. Gene Ontology enrichment analysis of the Bur0 accession transcripts that are enclosed in lost regions, including copy number variation and zero coverage reference genome portions (P = process, F = function and C = cellular component). (DOC 215 kb)

## Abbreviations

CNV: copy number variation
GUI: graphical user interface
GWAS: genome-wide association study
PAV: presence/absence variation
SNP: single nucleotide polymorphism
