# Supplementary figures and images for: Altools: a user friendly NGS data analyser
Source: Biol Direct. 2016 Feb 17;11:8. doi: 10.1186/s13062-016-0110-0 (PMC4756442; doi:10.1186/s13062-016-0110-0)

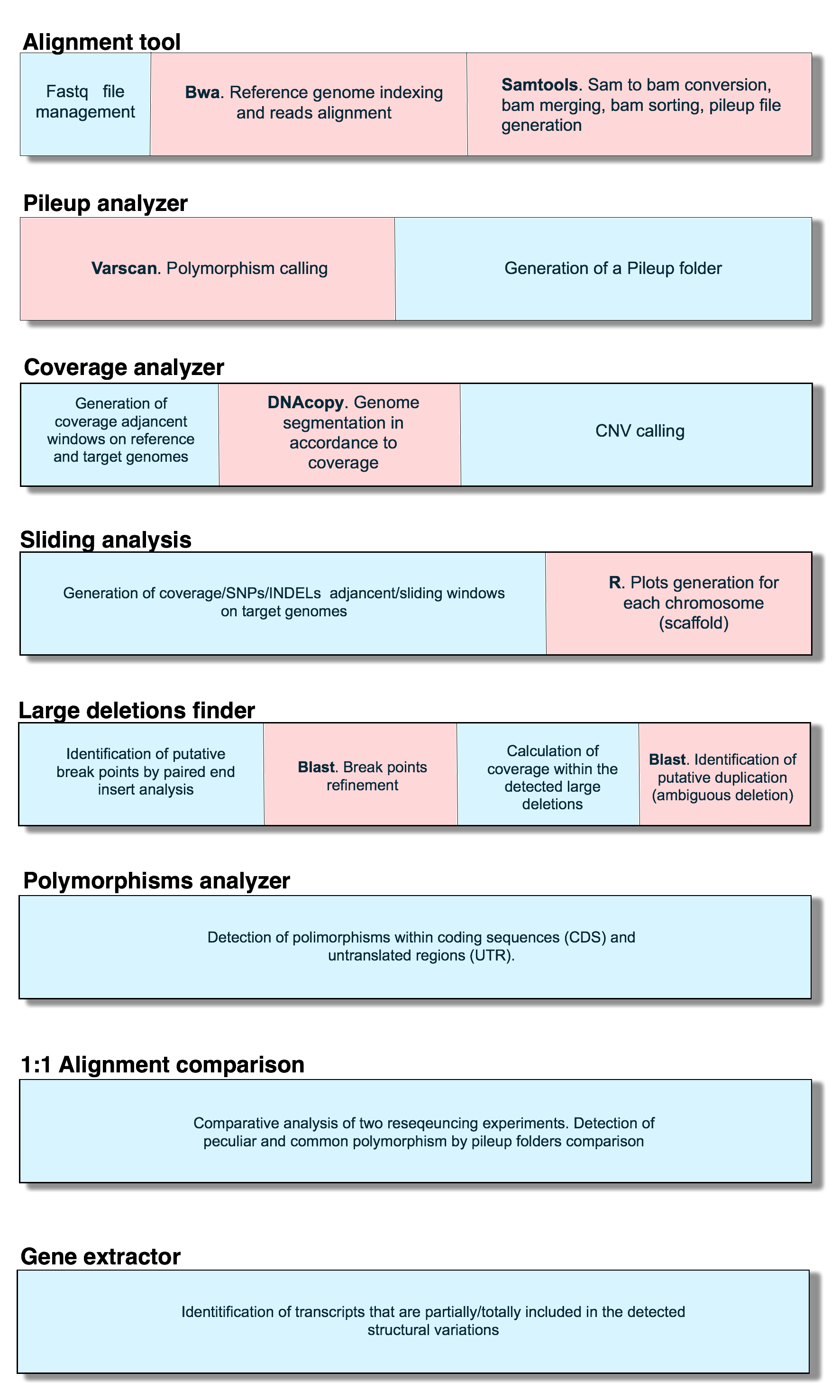

Supplement: Additional file 1: Figure S1. — Flowchart describing the eight Altools modules. Blue portions represent novel algorithms, whereas red portions represent third-party embedded software. (DOC 21 kb) [file 13062_2016_110_MOESM1_ESM.tiff]

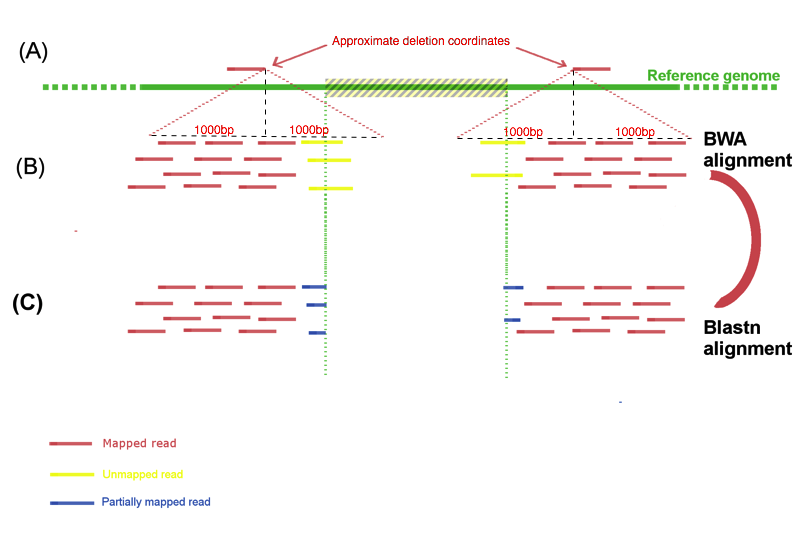

Supplement: Additional file 3: Figure S2. — Pipeline for the identification of deletion breakpoints. (a) Approximate deletion boundaries are inferred by detecting mapped paired-end reads that align at a distance that is not compatible with the expected insert. Overlapping sets of improperly-mapped mates (e.g. possibly underlining the same deletion) are merged at this stage. (b) A 2000-bp range is selected in the reference genome at each of the found deletion boundaries (deletion start ± 1000 bp and deletion end ± 1000 bp). Reads that are mapped within these regions are extracted from the alignment file together with the corresponding unmapped mates. (c) BLASTn is used to map reads identified at point (b) onto the reference genome and deletion breakpoints are inferred by the position of the detected partial alignments. (DOC 21 kb) [file 13062_2016_110_MOESM3_ESM.tiff]

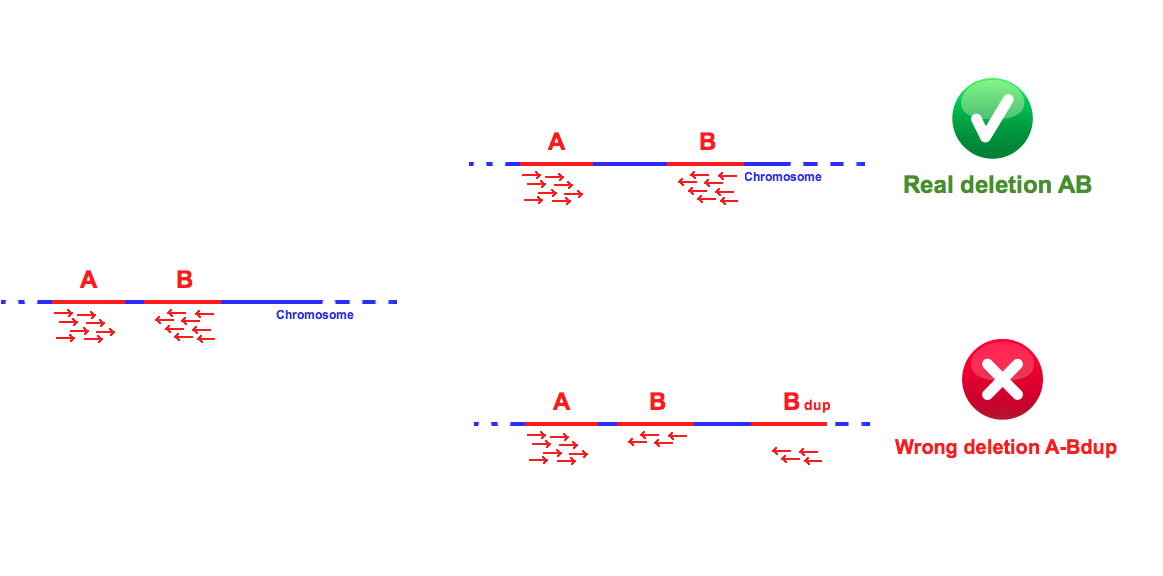

Supplement: Additional file 4: Figure S3. — Possible duplication interference affecting the correct identification of a large deletion. In a real deletion, reads mapping to the genomic portion A have their mates mapped to portion B at a distance that is not compatible with their library insert size. However, if a deletion did not occur between A and B, but rather B is duplicated somewhere upstream within the same chromosome, then reads mapping to A may have their mates mapped either in B or in Bdup. Mate pairs aligning in the portions A–Bdup will feature a mapping distance that is not compatible with their insert and, in this case, a deletion may be erroneously called. (DOC 21 kb) [file 13062_2016_110_MOESM4_ESM.tiff]

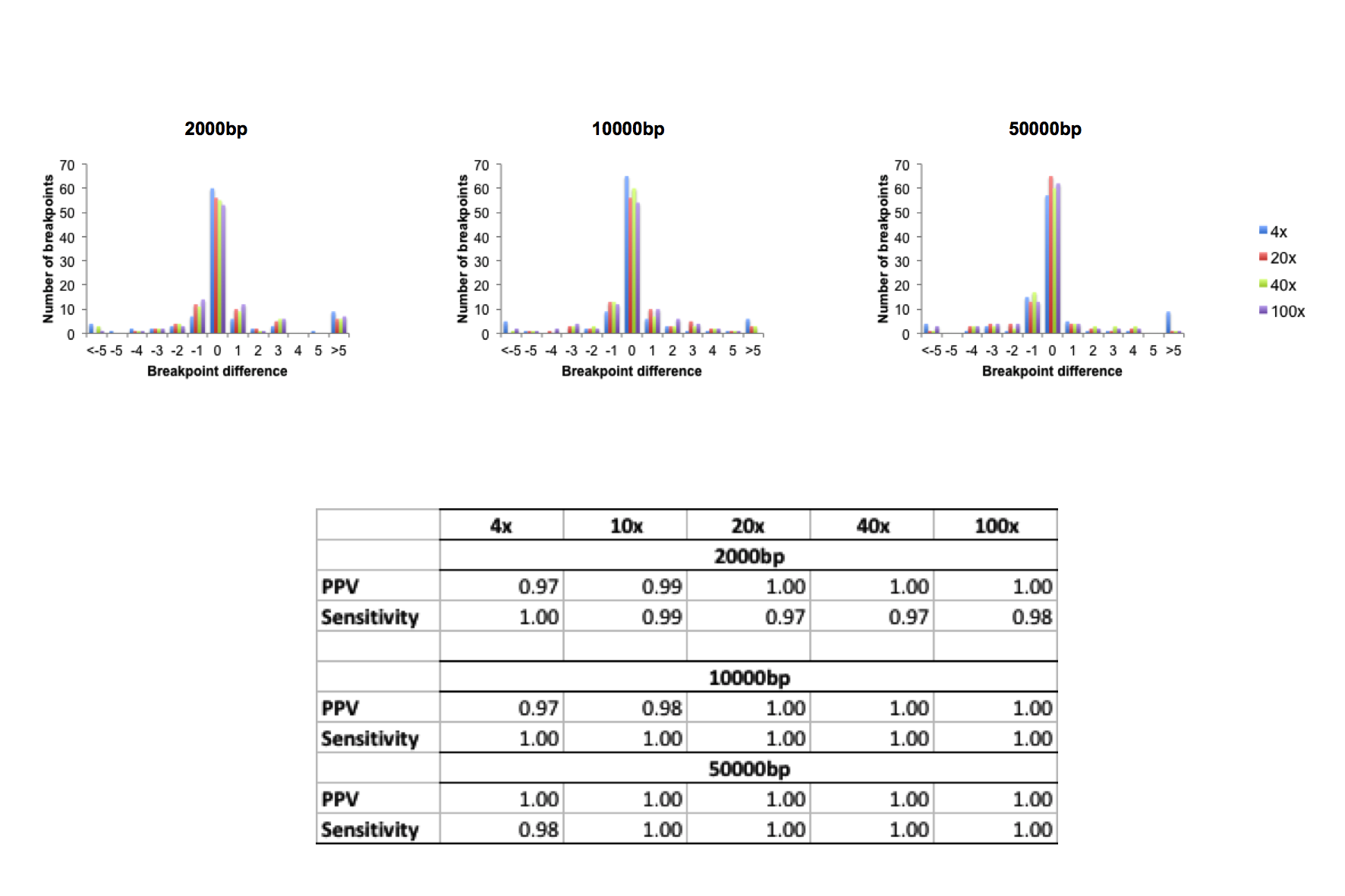

Supplement: Additional file 6: Figure S4. — Distribution of the differences (PPV and sensitivity) between detected and expected breakpoint positions derived from Large deletion finder analysis of the simulated reads dataset (coverage 4x, 20x, 40x and 100x) with three large deletion sizes (2000, 10000 and 50000 bp). (DOC 21 kb) [file 13062_2016_110_MOESM6_ESM.tiff]

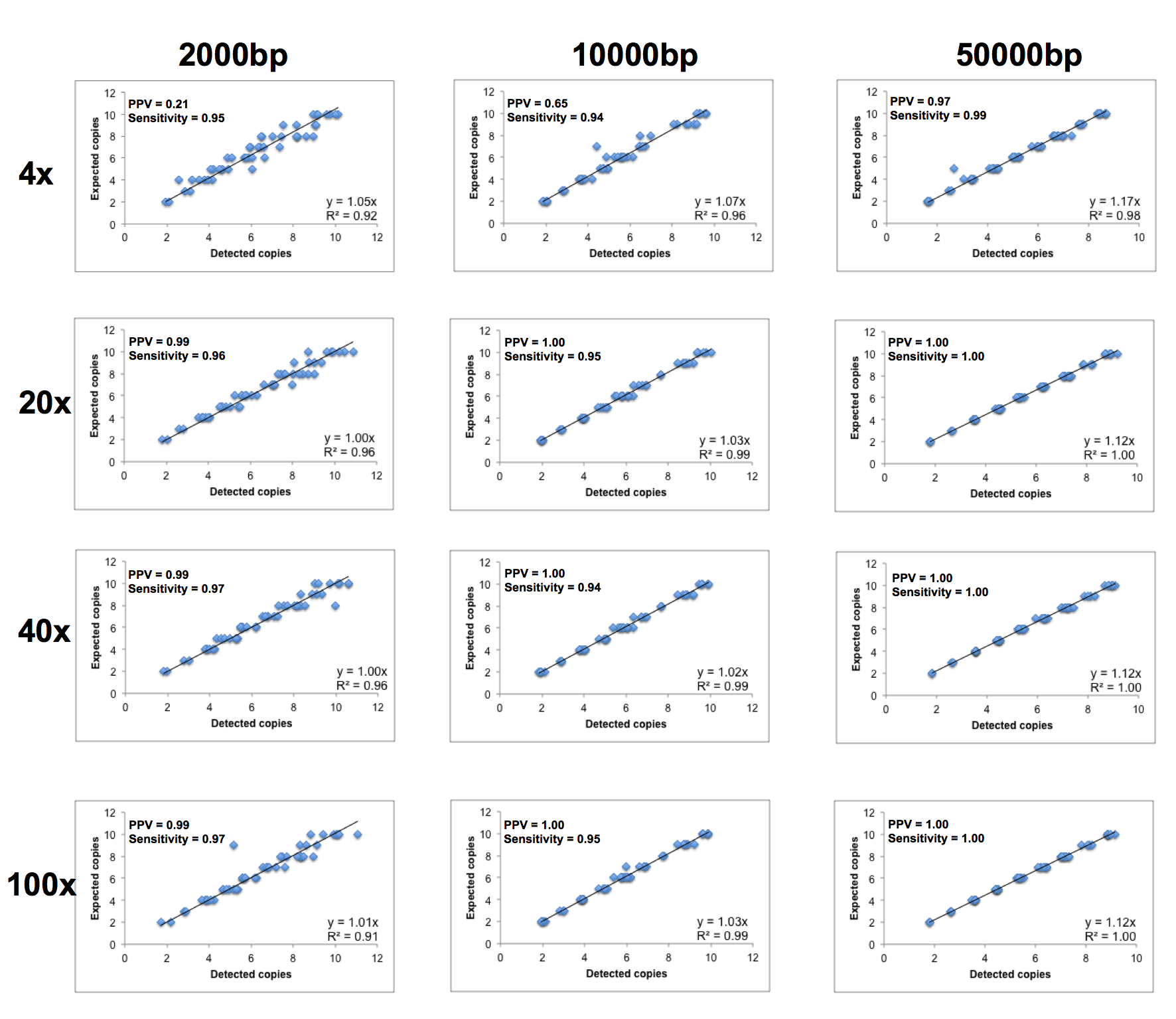

Supplement: Additional file 7: Figure S5. — Scatterplot showing differences (PPV and sensitivity) between detected and expected copy numbers calculated by the Coverage analyser tool on simulated reads datasets (coverage 4x, 20x, 40x and 100x) and three duplications sizes (2000, 10000 and 50000 bp). (DOC 21 kb) [file 13062_2016_110_MOESM7_ESM.tiff]

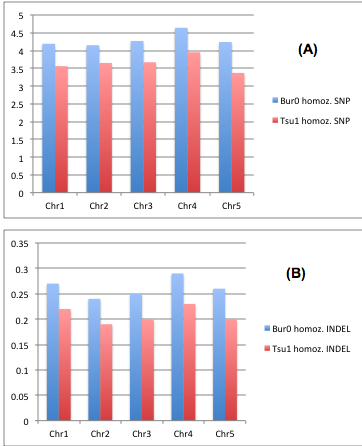

Supplement: Additional file 10: Figure S6. — Frequency of (A) SNPs and (B) indels in the alignment of Bur0 and Tsu1 sequences on the A. thaliana reference genome. (DOC 21 kb) [file 13062_2016_110_MOESM10_ESM.tiff]

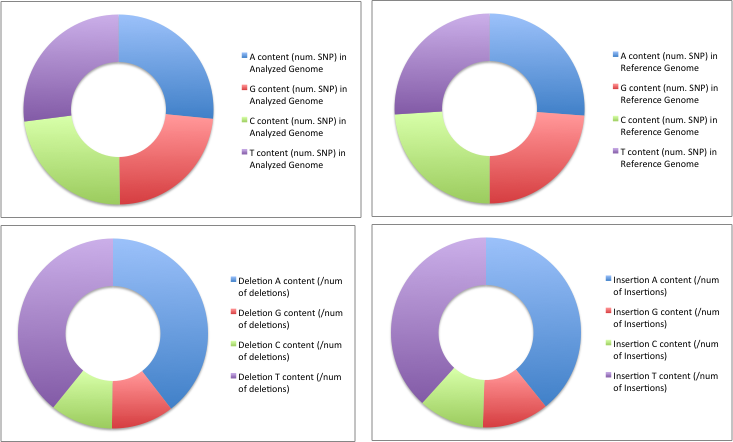

Supplement: Additional file 11: Figure S7. — (Top) Frequency of the four nucleotides in the reference and target genomes at a polymorphic site. (Bottom) Frequency of the four nucleotides among the inserted and deleted bases. (TIFF 142 kb) [file 13062_2016_110_MOESM11_ESM.tiff]

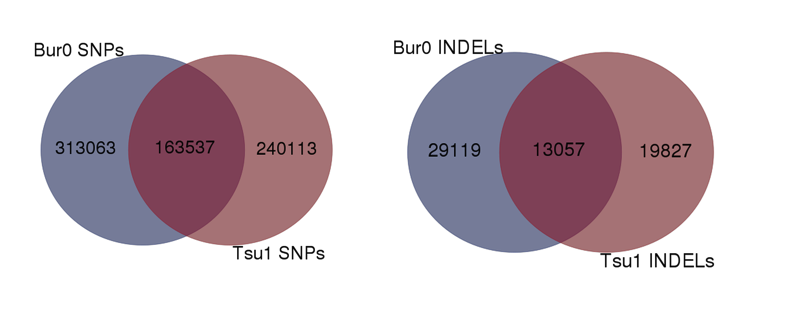

Supplement: Additional file 12: Figure S8. — Comparison of polymorphisms (SNPs and indels) found in the A. thaliana accessions Bur0 and Tsu1. (TIFF 68 kb) [file 13062_2016_110_MOESM12_ESM.tiff]
