# Supplementary material for: Altools: a user friendly NGS data analyser
Source: Biol Direct. 2016 Feb 17;11:8. doi: 10.1186/s13062-016-0110-0 (PMC4756442; doi:10.1186/s13062-016-0110-0)
Supplement: Additional file 2: Table S1. — Sequence read archive (SRA) experiments for A. thaliana accessions Bur0 and Tsu1 available at http://www.ncbi.nlm.nih.gov/sra. (DOC 209 kb) [file 13062_2016_110_MOESM2_ESM.doc]

| **Accession** | **SRA Experiment** |
| --- | --- |
| Bur0 | SRR013329 |
|  | SRR013330 |
|  | SRR013331 |
|  | SRR013332 |
|  | SRR013333 |
|  |  |
| Tsu1 | SRR013334 |
|  | SRR013335 |
|  | SRR013336 |
|  | SRR013337 |
|  | SRR013338 |
