# Supplementary material for: Altools: a user friendly NGS data analyser
Source: Biol Direct. 2016 Feb 17;11:8. doi: 10.1186/s13062-016-0110-0 (PMC4756442; doi:10.1186/s13062-016-0110-0)
Supplement: Additional file 5: Table S2. — Pileup analyser parameters to detect the simulated polymorphisms in the A. thaliana genome with different reference coverage values. (DOC 207 kb) [file 13062_2016_110_MOESM5_ESM.doc]

| **Coverage** | **4x** | **>= 10x** |
| --- | --- | --- |
| Average quality cutoff | 0 | 0 |
| Reference coverage cutoff | 2 | 5 |
| Minimum allele frequency | 0.1 | 0.1 |
| SNP/INDEL pValue | 0.05 | 0.05 |
| SNP/indel minimum coverage | 3 | 5 |
| SNP/INDEL minium reads | 2 | 3 |
