# Supplementary material for: Altools: a user friendly NGS data analyser
Source: Biol Direct. 2016 Feb 17;11:8. doi: 10.1186/s13062-016-0110-0 (PMC4756442; doi:10.1186/s13062-016-0110-0)
Supplement: Additional file 8: Table S3. — Benchmark of Altools for the detection of copy number variations (CNVs) and large deletions. The Coverage analyser module was compared to CNVseq [23] by testing its performance on the simulated A. thaliana genome with 10x coverage and three CNV segment sizes (2000, 10,000 and 50,000 bp). Default parameters were used in CNVseq except the window size (−−window-size 50) for the sake of uniformity with the Altools settings. The Large deletions finder module was compared to Pindel [10] by testing its performance on the simulated A. thaliana genome with 10x coverage and three deleted segment sizes (2000, 10,000 and 50,000 bp). To compare the software platforms under equivalent conditions, Pindel was set to output only deletions (−r false -t false -l false) while setting all the remaining parameters to their default values (for the detection of 50,000-bp deletions the flag –x 6 was added). Benchmarking was carried out on a server equipped with an Intel(R) Xeon(R) CPU X5660 working at 2.80 GHz. (DOC 21 kb) [file 13062_2016_110_MOESM8_ESM.doc]

| **Structural variation** | **Statistics** | **Tool** | **2000bp** | **10000bp** | **50000bp** |
| --- | --- | --- | --- | --- | --- |
| **CNV** | **Execution tme (min)** | Altools | 26 | 26 | 30 |
|  |  | CNV-seq | 642 | 706 | 940 |
|  |  |  |  |  |  |
|  | **PPV** | Altools | 0.72 | 0.96 | 1.00 |
|  |  | CNV-seq | 0.98 | 0.96 | 1 |
|  |  |  |  |  |  |
|  | **Sensitivity** | Altools | 0.95 | 0.94 | 1.00 |
|  |  | CNV-seq | 0.94 | 0.88 | 0.95 |
|  |  |  |  |  |  |
| **LD** | **Execution tme (min)** | Altools | 13 | 12 | 12 |
|  |  | Pindel | 90 | 102 | 761 |
|  |  |  |  |  |  |
|  | **PPV** | Altools | 0.99 | 0.98 | 1.00 |
|  |  | Pindel | 1.00 | 1.00 | 1.00 |
|  |  |  |  |  |  |
|  | **Sensitivity** | Altools | 0.99 | 1.00 | 1.00 |
|  |  | Pindel | 0.86 | 0.92 | 0.82 |
