# Supplementary material for: Altools: a user friendly NGS data analyser
Source: Biol Direct. 2016 Feb 17;11:8. doi: 10.1186/s13062-016-0110-0 (PMC4756442; doi:10.1186/s13062-016-0110-0)
Supplement: Additional file 9: Table S4. — G|C bias in the Bur0 and Tsu1 Illumina NGS datasets. (DOC 21 kb) [file 13062_2016_110_MOESM9_ESM.doc]

| **Chromosome name** | **Chr1** | **Chr2** | **Chr3** | **Chr4** | **Chr5** | **Average** |
| --- | --- | --- | --- | --- | --- | --- |
| **Bur0** |  |  |  |  |  |  |
| *G|C aver. Coverage* | 23.0 | 23.2 | 23.5 | 22.9 | 23.1 | 23.1 |
| *A|T aver. Coverage* | 21.4 | 21.4 | 21.8 | 21.3 | 21.5 | 21.5 |
|  |  |  |  |  |  |  |
| **Tsu1** |  |  |  |  |  |  |
| *G|C aver. Coverage* | 17.9 | 18.3 | 18.5 | 18.1 | 18.2 | 18.2 |
| *A|T aver. Coverage* | 16.5 | 16.7 | 16.9 | 16.6 | 16.7 | 16.7 |
