# Supplementary material for: Altools: a user friendly NGS data analyser
Source: Biol Direct. 2016 Feb 17;11:8. doi: 10.1186/s13062-016-0110-0 (PMC4756442; doi:10.1186/s13062-016-0110-0)
Supplement: Additional file 13: Table S5. — Gene Ontology enrichment analysis of the Bur0 accession transcripts that are enclosed in gained regions (P = process and F = function). (DOC 21 kb) [file 13062_2016_110_MOESM13_ESM.doc]

| **GO term** | **Ontology** | **Description** | **p-value** | **FDR** |
| --- | --- | --- | --- | --- |
| GO:0022904 | P | respiratory electron transport chain | 9.40E-10 | 5.60E-08 |
| GO:0006119 | P | oxidative phosphorylation | 5.10E-10 | 5.60E-08 |
| GO:0006091 | P | generation of precursor metabolites and energy | 1.70E-09 | 6.70E-08 |
| GO:0045333 | P | cellular respiration | 7.10E-08 | 1.70E-06 |
| GO:0015980 | P | energy derivation by oxidation of organic compounds | 7.10E-08 | 1.70E-06 |
| GO:0022900 | P | electron transport chain | 8.60E-08 | 1.70E-06 |
| GO:0055114 | P | oxidation reduction | 9.20E-06 | 0.00016 |
| GO:0016310 | P | phosphorylation | 0.0002 | 0.003 |
| GO:0006796 | P | phosphate metabolic process | 0.0004 | 0.0048 |
| GO:0006793 | P | phosphorus metabolic process | 0.0004 | 0.0048 |
| GO:0046483 | P | heterocycle metabolic process | 0.00073 | 0.008 |
| GO:0016491 | F | oxidoreductase activity | 1.30E-06 | 3.20E-05 |
| GO:0015078 | F | hydrogen ion transmembrane transporter activity | 1.10E-06 | 3.20E-05 |
| GO:0015077 | F | monovalent inorganic cation transmembrane transporter activity | 2.10E-06 | 3.50E-05 |
| GO:0022890 | F | inorganic cation transmembrane transporter activity | 1.70E-05 | 0.00022 |
| GO:0009055 | F | electron carrier activity | 0.00065 | 0.0064 |
| GO:0003735 | F | structural constituent of ribosome | 0.0011 | 0.0082 |
| GO:0008324 | F | cation transmembrane transporter activity | 0.0012 | 0.0082 |
| GO:0005198 | F | structural molecule activity | 0.0044 | 0.024 |
| GO:0015075 | F | ion transmembrane transporter activity | 0.0042 | 0.024 |
