# Supplementary material for: Altools: a user friendly NGS data analyser
Source: Biol Direct. 2016 Feb 17;11:8. doi: 10.1186/s13062-016-0110-0 (PMC4756442; doi:10.1186/s13062-016-0110-0)
Supplement: Additional file 14: Table S6. — Gene Ontology enrichment analysis of the Bur0 accession transcripts that are enclosed in lost regions, including copy number variation and zero coverage reference genome portions (P = process, F = function and C = cellular component). (DOC 215 kb) [file 13062_2016_110_MOESM14_ESM.doc]

| **GO term** | **Ontology** | **Description** | **p-value** | **FDR** |
| --- | --- | --- | --- | --- |
| GO:0006915 | P | apoptosis | 6.70E-09 | 7.60E-07 |
| GO:0012501 | P | programmed cell death | 4.20E-07 | 2.40E-05 |
| GO:0008219 | P | cell death | 1.80E-06 | 4.20E-05 |
| GO:0016265 | P | death | 1.80E-06 | 4.20E-05 |
| GO:0006952 | P | defense response | 1.20E-06 | 4.20E-05 |
| GO:0045087 | P | innate immune response | 6.00E-05 | 1.10E-03 |
| GO:0006955 | P | immune response | 9.30E-05 | 1.40E-03 |
| GO:0002376 | P | immune system process | 9.50E-05 | 1.40E-03 |
| GO:0004888 | F | transmembrane receptor activity | 1.50E-07 | 1.50E-05 |
| GO:0004872 | F | receptor activity | 9.50E-07 | 4.80E-05 |
| GO:0004871 | F | signal transducer activity | 2.80E-04 | 7.00E-03 |
| GO:0060089 | F | molecular transducer activity | 2.80E-04 | 7.00E-03 |
| GO:0012505 | C | endomembrane system | 5.20E-15 | 2.80E-13 |
